# Authors’ Response to Peer Reviews of “Checklist Approach to Developing and Implementing AI in Clinical Settings: Instrument Development Study”

**DOI:** 10.2196/69537

**Published:** 2025-02-20

**Authors:** Ayomide Owoyemi, Joanne Osuchukwu, Megan E Salwei, Andrew Boyd

**Affiliations:** 1 Department of Biomedical and Health Informatics, University of Illinois Chicago, 1919 W Taylor, Chicago, IL, 60612, United States, 1 3129782703; 2 College of Medicine, University of Cincinnati, Cincinnati, OH, United States; 3 Department of Biomedical Informatics, Vanderbilt University Medical Center, Nashville, TN, United States

**Keywords:** artificial intelligence, machine learning, algorithm, analytics, AI deployment, human-AI interaction, AI integration, checklist, clinical workflow, clinical setting, literature review

*This is the authors’ response to peer-review reports for “Checklist Approach to Developing and Implementing AI in Clinical Settings: Instrument Development Study.”*

## Round 1 Review

### Anonymous [1]

*The paper [* *2* *] presents the Clinical Artificial Intelligence (AI) Sociotechnical Framework (CASoF), a structured approach to guide the planning, design, development, and implementation of AI systems in health care settings. The framework is designed to address the gap between technical performance and sociotechnical factors that are essential for successful AI deployment in clinical environments.*

*The authors conducted a literature synthesis and a modified Delphi study involving global health care professionals to develop and refine the CASoF checklist. The checklist emphasizes the importance of considering the value proposition, data integrity, human-AI interaction, technical architecture, organizational culture, and ongoing support and monitoring, to ensure that AI tools are not only technologically sound but also practically viable and socially adaptable within clinical settings.*

*The study found that the successful integration of AI in health care depends on a balanced focus on both technological advancements and the sociotechnical environment of clinical settings. The CASoF represents a step forward in bridging this divide, offering a holistic approach to AI deployment that is mindful of the complexities of health care systems. The checklist aims to facilitate the development of AI tools that are effective, user-friendly, and seamlessly integrated into clinical workflows, ultimately enhancing patient care and health care outcomes.*

*The authors acknowledge some limitations of the study, such as the need for continuous refinement of the CASoF through iterative feedback and broader engagement with more stakeholders. Future research should aim to include an even wider array of perspectives, particularly from underrepresented regions and specialties, to enhance the framework’s comprehensiveness and applicability.*

*Overall, the paper provides a valuable contribution to the field of AI in health care by offering a practical and comprehensive approach to the development and implementation of AI systems in clinical settings.*

### Reviewer AE [3]

#### General Comments

*This paper presents the Clinical Artificial Intelligence (AI) Sociotechnical Framework (CASoF), a checklist intended to support the development and implementation of AI systems in health care settings. The framework is built on a comprehensive literature review and a modified Delphi study involving health care professionals globally. The manuscript addresses a significant gap in the integration of AI by emphasizing the importance of sociotechnical considerations alongside technical aspects.*

#### Specific Comments

##### Major Comments

*1. Clarity and structure: The manuscript could benefit from clearer explanations, particularly in the methodology section. The description of the Delphi study and literature synthesis is dense and may be difficult for readers to follow. Consider breaking down complex sentences and using more straightforward language.*

Response: Thank you for this; we have addressed and improved on the clarity and description of the methodology section as requested.

*2. Methodological rigor: The manuscript lacks details on the selection process for Delphi panelists and the exact methods used for data analysis. Transparency in these areas would significantly strengthen the paper. Additionally, clarify how the Delphi method was modified and the rationale behind these modifications.*

Response: We have addressed the selection process and what the modification of the Delphi process involves.

*3. Literature review and contextualization: The discussion section could benefit from a more critical comparison between the CASoF and existing frameworks. While the manuscript mentions other frameworks, it does not fully explore their limitations or how the CASoF overcomes these challenges. Expanding this discussion would provide a stronger justification for the CASoF’s novelty and utility.*

Response: We have added important comparisons with other existing frameworks/checklist and what utility the Clinical Artificial Intelligence (AI) Sociotechnical Framework (CASoF) has over them.

*4. Checklist practicality: While the checklist is comprehensive, its length and complexity may hinder practical adoption. Consider providing a condensed version for quick reference and include examples of how the checklist can be applied in real-world scenarios.*

Response: The application of the checklist in a real-world scenario has been highlighted. We appreciate the suggestion on providing a condensed version; however, we will retain the checklist in its present state and level. We created an online version to make the application easier [4].

*5. Ethical considerations and bias mitigation: The manuscript discusses ethical considerations but lacks specific strategies for addressing these issues within the CASoF. Expanding this discussion would enhance the manuscript’s comprehensiveness.*

Response: The checklist highlights specific questions that addresses ethical considerations; this has also been better highlighted in the manuscript.

##### Minor Comments

*6. Typographical and grammatical errors: There are minor typographical and grammatical errors throughout the manuscript that should be corrected. For instance, phrases like “revised and edited” could be simplified to “revised” for conciseness.*

Response: Thanks for this comment; this has been corrected.

*7. Tables and figures formatting: The tables summarizing the Delphi study results are helpful but could be more effectively formatted. Using shading or color coding to distinguish between different stages or domains would improve clarity and ease of interpretation.*

Response: Thanks, this is well noted. The final formatting would be more of a decision of the publisher.

*8. Recent references: Some references in the manuscript are relatively old, which is less ideal for a rapidly evolving field like AI. Where possible, the manuscript should incorporate more recent literature to support its claims and demonstrate the ongoing relevance of the topic.*

Response: The references for the articles were selected based on their relevance to the topic.

### Reviewer AP [5]

#### General Comments

*This paper...is a very cohesive approach to establishing a framework for the implementation of artificial intelligence (AI).*

#### Specific Comments

##### Major Comments

*1. Ideally there should be information on the demographics of the expert panel.*

*2. Please add comments regarding equitable access for these technologies.*

Response: We did not collect demographic data for the panelists except their professions.

### Reviewer BH [6]

#### General Comments

*Using artificial intelligence (AI) to add social and domain-specific steps to clinical trials is innovative. My only comment is whether the number of stages or the checklist changes if the shortlisted panelists change.*

Response: This change does not affect the number of changes. The process ends when consensus is reached.

#### Specific Comments

##### Major Comments

*1. I am unsure if having 38 (expert) panelists is good enough to have a robust framework.*

Response: Nasa et al [7] highlighted that a panel of 30‐50 is considered optimum for a Delphi study.

### Anonymous [8]

#### General Comments

*This paper construct a checklist to support the development and implementation of artificial intelligence (AI) in clinical settings. I only have some minor comments.*

#### Minor Comments

*1. Comparison with existing checklists: Please add a comparison with some of the existing checklists.*

Response: Thank you for this; we have added the necessary comparisons.

*2. Inconsistency in the number of studies: The authors initially stated that they included 20 studies, but later mentioned 23. Please clarify.*

Response: This has been corrected. There were 19 studies, 3 were excluded, and then 4 were added, which gives a final total of 20.

*3. Appendix visibility: The appendix is not visible.*

Response: This has been corrected.

*4. Abbreviation consistency: The abbreviation “IQR” appears multiple times. Please ensure it is clearly defined and used consistently.*

Response: This has been corrected. Thanks.

### Anonymous [9]

*This paper introduces the Clinical Artificial Intelligence (AI) Sociotechnical Framework (CASoF), a checklist developed through a literature synthesis and refined by a Modified Delphi study. It aims to guide the development and implementation of AI in clinical settings, focusing on the integration of both technological performance and sociotechnical factors. The framework addresses gaps in existing frameworks by emphasizing not only technical specifications but also the broader sociotechnical dynamics essential for successful AI deployment in health care.*

*New approaches to reporting AI in clinical settings are crucial as AI becomes more integrated into clinical practice. However, the paper needs to address the “black box” dilemma more thoroughly. This refers to the opaque nature of AI algorithms, where the decision-making process is not easily interpretable by clinicians, leading to trust and transparency issues. Additionally, while the CASoF checklist is a valuable tool, it would benefit from a more detailed comparison to established frameworks like TRIPOD (Transparent Reporting of a Multivariable Prediction Model for individual Prognosis or Diagnosis), which has been widely used in developing and validating clinical prediction models. Discussing how the CASoF complements or improves upon TRIPOD would strengthen the paper’s contributions.*

*I suggest adding a paragraph discussing the potential roles of AI when integrated into hospital electronic health record (EHR) systems. AI could be used for the development of advanced diagnostic and prognostic tools by analyzing real-time patient data. Integration with EHRs could enhance decision-making, providing predictive analytics at the point of care and improving patient outcomes. This would help explore the broader clinical impact of AI beyond just technical integration, addressing its potential for continuous learning and optimization in health care settings.*

Response: Thanks for your review, this is well noted.

## Abbreviations

AI: artificial intelligence
CASoF: Clinical Artificial Intelligence Sociotechnical Framework
